# The Ratio of ADP- to TRAP-Induced Platelet Aggregation Quantifies P2Y_12_-Dependent Platelet Inhibition Independently of the Platelet Count

**DOI:** 10.1371/journal.pone.0149053

**Published:** 2016-02-17

**Authors:** Christoph B. Olivier, Melanie Meyer, Hans Bauer, Katharina Schnabel, Patrick Weik, Qian Zhou, Christoph Bode, Martin Moser, Philipp Diehl

**Affiliations:** 1 Heart Center Freiburg University, Cardiology and Angiology I, Freiburg—Bad Krozingen, Germany; 2 Munich University Hospital, Institute and Outpatient Clinic for Occupational, Social, and Environmental Medicine, Munich, Germany; University of Leuven, BELGIUM

## Abstract

**Objective:**

This study aimed to assess the association of clinical factors with P2Y_12_-dependent platelet inhibition as monitored by the ratio of ADP- to TRAP-induced platelet aggregation and conventional ADP-induced aggregation, respectively.

**Background:**

Controversial findings to identify and overcome high platelet reactivity (HPR) after coronary stent-implantation and to improve clinical outcome by tailored anti-platelet therapy exist. Monitoring anti-platelet therapy *ex vivo* underlies several confounding parameters causing that *ex vivo* platelet aggregation might not reflect *in vivo* platelet inhibition.

**Methods:**

In a single centre observational study, multiple electrode aggregometry was performed in whole blood of patients after recent coronary stent-implantation. Relative ADP-induced aggregation (r-ADP-agg) was defined as the ratio of ADP- to TRAP- induced aggregation reflecting the individual degree of P2Y_12_-mediated platelet reactivity.

**Results:**

Platelet aggregation was assessed in 359 patients. Means (± SD) of TRAP-, ADP-induced aggregation and r-ADP-agg were 794 ± 239 AU*min, 297 ± 153 AU*min and 37 ± 14%, respectively. While ADP- and TRAP-induced platelet aggregation correlated significantly with platelet count (ADP: r = 0.302; p<0.001; TRAP: r = 0.509 p<0.001), r-ADP-agg values did not (r = -0.003; p = 0.960). These findings were unaltered in multivariate analyses adjusting for a range of factors potentially influencing platelet aggregation. The presence of an acute coronary syndrome and body weight were found to correlate with both ADP-induced platelet aggregation and r-ADP-agg.

**Conclusion:**

The ratio of ADP- to TRAP-induced platelet aggregation quantifies P2Y_12_-dependent platelet inhibition independently of the platelet count in contrast to conventional ADP-induced aggregation. Furthermore, r-ADP-agg was associated with the presence of an acute coronary syndrome and body weight as well as ADP-induced aggregation. Thus, the r-ADP-agg is a more valid reflecting platelet aggregation and potentially prognosis after coronary stent-implantation in P2Y_12_-mediated HPR than conventional ADP-induced platelet aggregation.

## Introduction

After coronary stent-implantation, guidelines recommend a dual anti-platelet therapy with ASA and P2Y_12_-receptor antagonists. Clopidogrel is the most widely used P2Y_12_-antagonist. Clinical trials have demonstrated that high platelet reactivity (HPR) after clopidogrel administration increases the risk of recurrent atherothrombotic events [1]. However, controversial findings to identify and overcome HPR after coronary stent-implantation and to improve clinical outcome by tailored anti-platelet therapy exist [2–8]. Patients with HPR are commonly identified by ADP-induced platelet aggregometry, performed by VerifyNow^®^ assay and multiple electrode aggregometry (MEA) [9]. However, *ex vivo* platelet aggregation is potentially influenced by biasing factors (e.g. parameters of the haemogram) making it hard to transfer results from platelet aggregometry on *in vivo* platelet function [10]. It has been suggested that activating platelets via *T*hrombin *R*eceptor *A*ctivating *P*eptide (TRAP) and the thrombin receptor might serve as an “internal control” for ADP-induced platelet inhibition [11, 12], to selectively indicate the degree of P2Y_12_–mediated platelet reactivity.

Recently, our group showed that the ratio of ADP- to TRAP-induced platelet aggregation (r- ADP-agg) is a valuable tool reflecting a patient’s individual degree of P2Y_12_–mediated platelet reactivity and that a high r-ADP-agg is associated with an increased mortality in patients after coronary stent-implantation on clopidogrel [13, 14]. This data suggest that r-ADP-agg might predict the clinical outcome of patients after percutaneous coronary intervention more precisely than ADP-induced platelet aggregation alone.

The aim of the present study was to further investigate the clinical utility of r-ADP-agg by analysing clinical factors influencing P2Y_12_-dependent platelet reactivity as monitored by r-ADP-agg in comparison to conventional ADP-induced aggregation.

## Methods

### Study design and clinical characteristics

Data of this study were collected during an observational single centre study in which the anti-platelet effect of clopidogrel in patients after coronary stent implantation had been investigated. The study was performed in accordance with the Helsinki declaration and was approved by the ethics committee of the Albert-Ludwigs-University Freiburg, Germany (registry number 183/07). All patients gave their written informed consent prior to study participation. Patients were included prospectively in the Department of Cardiology and Angiology at the Heart Center of the University of Freiburg from 2007 to 2011. All patients received 100 mg ASA per day and 75 mg clopidogrel after a loading dose of 300 mg (at least 24 hours before platelet aggregation assay) or 600 mg (at least 12 hours before platelet aggregation assay). The washout period for the GPIIb/IIIa antagonist eptifibatid was 12 hours. Patients with coagulation disorders such as antiphospholipid syndrome were excluded.

### Blood samples

Venous blood was collected using a 21 G butterfly needle (Safety-Multifly^®^-Set, Sarstedt, Nümbrecht, Germany) to a final concentration (Fc) of >15 μg/ml r-hirudin (SARSTEDT Monovetten, Nümbrecht, Germany). To prevent storage induced platelet activation, blood samples were analysed within the first two hours after venous puncture.

### Platelet aggregometry

Multiple electrode aggregometry (MEA, Roche Diagnostics, Switzerland) was performed in whole blood of patients after percutaneous coronary intervention (PCI) under anti-platelet medication as recently described [13, 14]. In order to assess the overall platelet aggregability, blood samples were stimulated with TRAP (Fc 32 μM). To specifically quantify the effect of P2Y_12_-inhibitors, whole blood was stimulated with ADP (Fc 6.4 μM). R-ADP-agg was defined as the ratio of ADP- to TRAP-induced platelet aggregation.

### Statistical analysis

Data are presented as numbers with frequencies for categorical variables and means with standard deviations (SD) for continuous variables. The Kolmogorov-Smirnov test was applied to assess normality. Group comparisons for normally distributed variables were performed with Student's t-test. In non-normally distributed parameters, the Mann-Whitney U test was used. Correspondingly, Pearson’s or Spearman’s coefficients were used for bivariate correlational analyses. All tests were 2-tailed and p values ≤0.05 were considered statistically significant. The association of covariates was tested by analyses of covariance (ANCOVA). To identify patient characteristics that show a significant association with aggregation markers even after mutual statistical adjustment (accounting for confounding effects), separate multiple regression models of the three aggregation markers including potentially relevant influencing available factors (following the review of Siller-Matula et al. [15]) as covariates were run on all patients with complete data. The final models reported here were then obtained by a backward elimination procedure removing all covariates with a p value >0.1 in a stepwise manner. Data were analysed with Prism 5.01 (GraphPad Software, La Jolla, California, USA) and SPSS 21.0.0.1 (SPSS Inc, Chicago, Illinois, USA).

## Results

### Sample characteristics

In total, platelet function was assessed in 359 patients. The mean of TRAP-induced aggregation, ADP-induced aggregation and r-ADP-agg was 794 ± 239 AU*min, 297 ± 153 AU*min and 37 ± 14% respectively. ADP- and TRAP-induced aggregation correlated significantly (r = 0.623, p<0.001), while r-ADP-agg correlated significantly with ADP-induced aggregation (r = 0.769, p<0.001) but not with TRAP-induced aggregation (r = 0.041, p = 0.041). Clinical baseline characteristics are shown in Table 1.

**Table 1:** Clinical baseline characteristics.

	n = 359
Age [years]	70 (12)
Male sex	245 (68)
Body weight [kg]	79 (16)
**Procedural data**	
Acute Coronary Syndrome	210 (58)
One-vessel CAD	72 (20)
Two-vessel CAD	86 (24)
Three-vessel CAD	201 (56)
Implanted stents	1.3 (0.7)
**Medical history**	
Previous MI	77 (21)
Severely reduced LV-function	31 (8.6)
Implanted cardioverter-defibrillator	15 (4.2)
Atrial fibrillation	65 (18)
Stroke/transient ischemic attack	40 (11)
Gastrointestinal bleeding	10 (2.8)
**Cardiovascular risk factors**	
History of smoking	148 (41)
Diabetes mellitus	106 (30)
Arterial hypertension	275 (77)
Family history of MI	85 (24)
Hyperlipidemia	213 (59)
**Laboratory data**	
WBC [10^3^/μl]	8.2 (2.8)
RBC [10^3^/μl]	4.5 (0.6)
Platelets [10^3^/μl]	230 (71)
Haemoglobin [g/dl]	13 (1.9)
CK [U/ml]	280 (710)
CRP [mg/l]	15 (26)
Cholesterol [mg/dl]	190 (43)
Creatinine [mg/dl]	1.1 (0.6)
**Comedication**	
ACE inhibitor	245 (68)
β blocker	312 (87)
Calcium antagonist	88 (25)
Diuretic	148 (41)
Statin	319 (89)
Proton-pump inhibitor	196 (55)
Oral anticoagulant	17 (4)

Data are n (%) or mean (SD).

## Aggregation Markers

**Fig 1:**
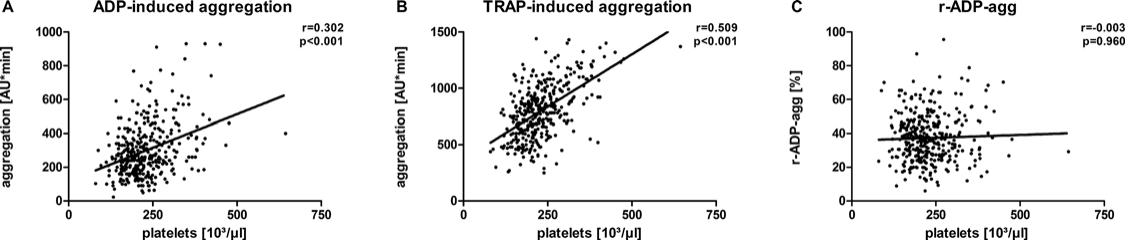
While ADP- and TRAP-induced platelet aggregation correlated significantly with platelet counts the r-ADP-agg values were independent of those.

While ADP- and TRAP-induced platelet aggregation correlated significantly with platelet counts (PC) (ADP: r = 0.302, p<0.001; TRAP: r = 0.509, p<0.001), the r-ADP-agg values were independent of those (r = -0.003; p = 0.960) (Fig 1). A significant correlation of white blood cell counts (WBC) with ADP- and TRAP-induced aggregation (ADP: r = 0.229, p<0.001; TRAP: r = 0.278, p<0.001) was observed while this was not found for r-ADP-agg (r = 0.083; p = 0.118). No significant correlation was found for all aggregation markers with red blood cell counts (RBC) (ADP: r = 0.021, p = 0.689; TRAP: r = -0.071, p = 0.183; r-ADP-agg: r = 0.071; p = 0.181). Furthermore, factors that might influence platelet reactivity after clopidogrel treatment were analysed following the description of Siller-Matula [15, 16] according to availability as shown in Table 2. Body weight was found to correlate significantly with both ADP-induced platelet aggregation (r = 0.143, p = 0.007) and r-ADP-agg (r = 0.157, p = 0.003), but not with TRAP-induced platelet aggregation. Nevertheless, it was found that TRAP-induced platelet aggregation correlated significantly with age (r = -0.105, p = 0.048) while this was not seen for ADP-induced aggregation or r-ADP-agg. Creatinine levels did not correlate significantly with any of the aggregation markers. Although CRP and cholesterol was not available in all patients (n = 245 and 129 respectively) no significant correlations were found.

**Table 2:** Association of patient characteristics with aggregation markers.

	ADP induced aggregation [AU*min]		TRAP-induced aggregation [AU*min]		r-ADP-agg [%]	
**Continuous variables**	**correlation**	**p-value**	**correlation**	**p-value**	**correlation**	**p-value**
WBC [10^3^/μl]	0.229	<0.001	0.278	<0.001	0.083	0.118
RBC [10^3^/μl]	0.021	0.689	-0.071	0.183	0.071	0.181
Age [years]	-0.098	0.066	-0.105	0.048	-0.016	0.762
Body weight [kg]	0.143	0.007	-0.020	0.704	0.157	0.003
CRP [mg/dl]	0.099	0.123	0.056	0.379	0.086	0.178
Cholesterol [mg/dl]	-0.106	0.229	-0.023	0.795	-0.063	0.475
Creatinine [mg/dl]	0.026	0.629	0.026	0.621	0.016	0.762
**Categorical variables**	**mean (SD)**	**p-value**	**mean (SD)**	**p-value**	**mean (SD)**	**p-value**
male vs.	305 (152) vs.	0.110	788 (239) vs.	0.313	39 (14) vs.	0.032
Female	288 (165)		815 (246)		35 (14)	
ACS vs.	323 (159) vs.	<0.001	801 (243) vs.	0.888	40 (13) vs.	<0.001
No ACS	266 (146)		790 (239)		34 (14)	
Severely reduced LV-function vs.	286 (146) vs.	0.757	776 (237) vs.	0.798	37 (15) vs.	0.812
Not severely reduced LV function	300 (158)		799 (242)		37 (14)	
Diabetes mellitus vs.	327 (183) vs.	0.120	835 (252) vs.	0.052	39 (15) vs.	0.301
no diabetes mellitus	287 (143)		781 (235)		37 (14)	
β-blocker vs.	305 (156) vs.	0.041	799 (237) vs.	0.757	38 (14) vs.	0.022
No β-blocker	264 (156)		183 (267)		33 (15)	
Calcium antagonist vs.	291 (171) vs.	0.324	797 (260) vs.	0.947	36 (14) vs.	0.229
No calcium antagonist	302 (152)		797 (235)		38 (14)	
PPI vs.	315 (169) vs.	0.062	821 (255) vs.	0.073	38 (15) vs.	0.437
No PPI	277 (136)		767 (220)		36 (13)	

In the analysis of categorical variables there was a trend towards a higher r-ADP-agg in male compared to female patients (p = 0.032). This observation is most likely explained by a higher body weight in men compared to women (mean male vs. female: 84 ± 14 vs 69 ± 15 kg; p<0.001). Consistent with this explanation, analysis of covariance (ANCOVA) showed that the difference between the hypothetical means adjusted for the covariate body weight was substantially smaller (hypothetical means for r-ADP-agg; male: 38% vs female 36%; p = 0.317). Furthermore, ADP-induced aggregation and r-ADP-agg were enhanced in patients presenting with an acute coronary syndrome (ACS) (effect size for ADP: r = 0.364, p<0.001; r-ADP-agg: r = 0.423, p<0.001) and under treatment with β-blockers (effect size for ADP: r = 0.262, p = 0.041; r-ADP-agg: r = 0.352, p = 0.022). Severely reduced left ventricular function, diabetes mellitus, the use of calcium antagonists or proton pump inhibitors were not associated significantly with aggregation markers.

Results of the multiple regression analyses to identify factors significantly associated with aggregations markers under mutual adjustment are presented in Table 3. Only parameters of covariates that were retained in the model of the respective aggregation marker during the backward elimination procedure are included in the table. Cholesterol and CRP were not included in the multiple regression analyses because these biomarkers were not available in all patients. Furthermore, their bivariate correlations with aggregation markers were quite weak (all |r| < 0.11).

**Table 3:** Factors significantly associated with aggregation markers in multiple regression analyses (all N = 351).

	ADP induced aggregation [AU*min]				TRAP-induced aggregation [AU*min]				r-ADP-agg [%]			
	Coef	95% CI	Std Coef	p	Coef	95% CI	Std Coef	p	Coef	95% CI	Std Coef	p
Intercept	-94.09	-197.64; 9.47	-	-	470.92	333.04; 608.81	-	-	19.67	11.69; 27.66	-	-
Platelets [10^3^/μl]	0.82	0.61; 1.04	0.37	<0.001	1.89	1.60; 2.19	0.55	<0.001	-	-	-	-
WBC [10^3^/μl]	-	-	-	-	-	-	-	-	-	-	-	-
RBC [10^3^/μl]	-	-	-	-	-	-	-	-	-	-	-	-
Age [years]	-	-	-	-	-1.78	-3.47; -0.10	-0.09	0.038	-	-	-	-
Body weight [kg]	1.40	0.45; 2.34	0.14	0.004	-	-	-	-	0.14	0.05; 0.23	0.15	0.003
Creatinine [mg/dl]	**-**	**-**	**-**	**-**	-	-	-	-	-	-	-	-
Male sex	-	-	-	-	-	-	-	-	-	-	-	-
Acute coronary syndrome	42.87	12.22; 73.51	0.14	0.006	-	-	-	-	5.90	2.99; 8.81	0.21	<0.001
Severely reduced LV func.	-	-	-	-	-	-	-	-	-	-	-	-
Diabetes mellitus	37.14	4.55; 69.72	0.11	0.026	52.47	6.42; 98.51	0.10	0.026	-	-	-	-
β-blocker	49.35	4.81; 93.89	0.11	0.030	-	-	-	-	4.05	-0.25; 8.36	0.10	0.065
Calcium antagonist	-	-	-	-	-	-	-	-	-	-	-	-
Proton pump inhibitor	27.20	-3.24; 57.63	0.09	0.080	-	-	-	-	-	-	-	-
R^2^	0.206				0.325				0.084			

Coef: Regression coefficient. CI: Confidence interval. Std coef: Standardized regression coefficient. Variables where no units are provided in brackets are binary. Models were run seperately with each of the three aggregation markers as the outcome, initially including all factors displayed in this table as covariates, and then removing covariates with p > 0.1 in a backward elimination procedure. Covariates where the coefficient is missing were removed from the respective model in this manner.

With regards to ADP-induced aggregation, there was no significant association between WBC and ADP-induced aggregation in the multiple regression models, in contrast to the bivariate correlation coefficient reported above. This pattern of results can be explained by a confounding effect of platelet count, as also evidenced by a significant bivariate correlation between PC and WBC (r = 0.315; p<0.001). In contrast, PC is retained in the model. In fact, it has the strongest association with ADP-induced aggregation among all patient characteristics, as evidenced by the standardized coefficient. Further relevant factors include body weight, the presence of ACS or diabetes, use of beta blockers and, to a lesser degree, of proton pump inhibitors.

Like ADP-induced aggregation, TRAP-induced aggregation is significantly predicted by diabetes and platelet count. Age is uniquely associated with TRAP, whereas other factors relevant in ADP-induced aggregation do not play a noticeable role. Thus, TRAP is associated with fewer characteristics than ADP-induced aggregation, however, the association with PC is so strong that the percentage of explained variation is 32.5%, as opposed to 20.6 in ADP-induced aggregation.

Finally, r-ADP-agg is significantly predicted by body weight, the presence of ACS, and the use of beta blockers. There is no significant difference between the sexes after adjustment for the other covariates, consistent with the results of the corresponding ANCOVA adjusting for body weight. Most importantly, r-ADP-agg is the only one of the aggregation markers not associated with platelet count, confirming the corresponding bivariate analyses. Of note, the strongest association of r-ADP-agg is with ACS (as measured by standardized coefficients), which highlights the potential clinical utility of this aggregation marker. The proportion of variation in r-ADP-agg explained by the examined patient characteristics (8.4%) is noticeably lower than that in the other markers.

## Discussion

The principal finding of this study is that r-ADP-agg does not depend on platelet count while conventional ADP-induced aggregation does.

Data published in 2011 by Gremmel et *al*. confirm our results: ADP-induced aggregation measured by MEA depends on the PC [17]. Interestingly, they found that this was only the case for MEA and not for other methods. The reason for this finding might be related to the underlying mechanism of MEA: activated by an agonist, the platelets adhere to the electrodes and cause the change of impedance. Higher platelet numbers might therefore cause higher changes of impedance [17].

Voisin et *al*. assessed platelet aggregation in 186 patients under dual anti-platelet therapy using VerifyNow [10]. They showed that ADP- and TRAP-induced platelet aggregation significantly decrease with increasing haematocrit or haemoglobin, whereas a ratio of ADP- to TRAP-induced aggregation does not which might be due to a parallel change of both markers. Nevertheless, they suggested that the ADP- to TRAP-induced ratio more precisely reflects P2Y_12_-mediated platelet aggregation and hence can be used to assess the effect of P2Y_12_-receptor inhibition. In the study of Voisin *et al*. data of platelet and white blood cell count and the influence on aggregation markers is not shown. Although, in the present study using MEA there was no association of the red blood cell count with aggregation markers, the findings of Voisin *et al*. confirm that platelet aggregometry might not be independent of haematological variables. Nevertheless, in contrast to the VerifyNow^®^ assay it has been suggested that r-ADP-agg measured by MEA might be more precise to predict clinical outcome than conventional ADP-induced aggregation alone [14].

Numerous factors can influence the extent of platelet inhibition by clopidogrel [15, 16]. Some of these factors are assay-dependent [13], which is also partly evidenced by the present study’s results. For example, presence of diabetes and proton pump inhibitor medication appeared to be more strongly associated with ADP-induced aggregation than with r-ADP-agg. Increased r-ADP-agg in men and the correlation of ADP-induced aggregation with WBC may be explained in terms of confounding by body weight and platelet count, respectively.

At the same time, however, the association between PC and r-ADP-agg remained very weak and non-significant in both bivariate and multivariate analyses, whereas there was a clearly detectable association between PC and ADP-induced aggregation which remained even after adjusting for a range of other patient characteristics. As PC can be considered a nuisance factor in the selective assessment of P2Y_12_-mediated platelet reactivity, r-ADP-agg may have higher clinical utility than conventional ADP-induced aggregation in the diagnosis of P2Y_12_-mediated HPR and associated thrombotic events. In the present analysis, approximately 10% of the variance in ADP-induced aggregation measurements could be explained by variations in patients’ PC (r^2^ = 0.302^2^ = 0.091) and could be considered nuisance variation leading to reduced precision in the quantification of individual P2Y_12_-mediated platelet reactivity. Eliminating this source of variation, as in r-ADP-agg, may thus lead to higher precision and higher power to detect the effects of prognostic and interventional factors on clinical outcomes. For example, our group recently showed that a high r-ADP-agg is associated with an increased mortality in patients after coronary stent-implantation and clopidogrel therapy [14]. Further clinical trials should evaluate if an adaption of P2Y_12_-inhibition monitored by r-ADP-agg can improve clinical outcome.

In the multiple regression model both, ADP-induced aggregation and r-ADP-agg were associated with the presence of an ACS setting and body weight. These factors had been described previously to be associated with HPR [15]. Interestingly, among all investigated patient characteristics, r-ADP-agg was most strongly associated with the presence of ACS.

TRAP is one of the strongest platelet activators and leads to pronounced platelet aggregation [18–20]. Badr et al. recently observed that platelet activation via thrombin receptors PAR-1 and PAR-4 is preserved in the majority of patients after platelet inhibition with clopidogrel. Nevertheless, it has been described that TRAP-induced platelet aggregation is slightly reduced in patients under treatment by thienopyridines [21]. However, Iyu et al. recently found that P2Y_12_-antagonists produced only minor additional inhibition of TRAP-induced aggregation and found no evidence that any of the P2Y_12_-antagonists act through other G-protein receptors including PAR1 [22]. Results from our group comparing different P2Y_12_-inhibitors showed that there are no differences of the TRAP-induced platelet aggregation in the corresponding medication group [13]. Although controversial findings exist on the influence of P2Y_12_-inhibitors TRAP-induced aggregation might be a reasonable internal control for patients under clopidogrel medication, as also stated by Gremmel et al. in 2010 [11, 12]. Nevertheless, in patients receiving co-medication that might influence TRAP-induced platelet aggregation such as vorapaxar the r-ADP-agg might not be applicable to monitor HPR after clopidogrel treatment [19, 23, 24].

## Study Limitations

The present study is based on a post-hoc analysis of observational data. Therefore, confounders cannot be excluded. As we only performed a single measurement of platelet reactivity in the steady state of clopidogrel intake, pharmacodynamic differences in the first hours are not displayed. Only one method of platelet aggregometry was performed.

## Conclusion

The ratio of ADP- to TRAP-induced platelet aggregation quantifies P2Y_12_-dependent platelet inhibition independently of the PC in contrast to conventional ADP-induced aggregation. Furthermore, r-ADP-agg was associated with the presence of an ACS and body weight as well as ADP-induced aggregation. Thus, the r-ADP-agg is a more valid reflecting platelet aggregation and potentially prognosis after coronary stent-implantation in P2Y_12_-mediated HPR than conventional ADP-induced platelet aggregation. Whether an adaption of P2Y_12_-antagonism monitored by r-ADP-agg can provide an additional clinical benefit needs to be evaluated in further trials.
